# Supplementary material for: Modified Clerodanes from the Essential Oil of Dodonea viscosa Leaves
Source: Molecules. 2020 Feb 14;25(4):850. doi: 10.3390/molecules25040850 (PMC7070720; doi:10.3390/molecules25040850)
Supplement: Supplementary file 1 [file molecules-25-00850-s001.pdf]

Article

Modified clerodanes from the essential oil of *Dodonea viscosa* leaves

Arnaud Marvilliers [1], Bertrand Illien [1], Emmanuelle Gros [1], Jonathan Sorres [2], Yoel Kashmann [3], Hermann Thomas [4], Jacqueline Smadja [1] and Anne Gauvin-Bialecki [1,*]

[1] Laboratoire de Chimie des Substances Naturelles et des Sciences des Aliments, Faculté des Sciences et Technologies, Université de la Réunion, 15 Avenue René Cassin, CS 92 003, 97 744 St Denis, Messag Cedex 9, La Réunion, France
[2] Service RMN, Institut de Chimie des Substances Naturelles, CNRS, 91 110 Gif-sur-Yvette, France
[3] School of Chemistry, Tel Aviv University, 69978 Tel Aviv, Israël
[4] Parc national de La Réunion, Secteur Nord, 165 allée des spinelles Bellepierre, 97400 St-Denis, France

* Correspondence: e-mail: anne.bialecki@univ-reunion.fr; Tel.: +262-262-93 81 97

Received: date; Accepted: date; Published: date

P2 **Figure S1.** ^{1}H NMR spectrum of **1** at 600 MHz in $CDCl_3$.
P2 **Figure S2.** ^{1}H NMR spectrum of **1** at 600 MHz in $CDCl_3$.
P3 **Figure S3.** ^{13}C NMR (APT) spectrum of **1** at 150 MHz in $CDCl_3$.
P3 **Figure S4.** ^{13}C NMR (APT) spectrum of **1** at 150 MHz in $CDCl_3$.
P4 **Figure S5.** HSQC NMR spectrum of **1** at 600 MHz in $CDCl_3$.
P4 **Figure S6.** HSQC NMR spectrum of **1** at 600 MHz in $CDCl_3$.
P5 **Figure S7.** HMBC NMR spectrum of **1** at 600 MHz in $CDCl_3$.
P5 **Figure S8.** HMBC NMR spectrum of **1** at 600 MHz in $CDCl_3$.
P6 **Figure S9.** HMBC NMR spectrum of **1** at 600 MHz in $CDCl_3$.
P7 **Figure S10.** NOESY NMR spectrum of **1** at 600 MHz in C_6D_6.
P7 **Figure S11.** NOESY NMR spectrum of **1** at 600 MHz in C_6D_6.
P8 **Figure S12.** (+)HRESIMS spectrum of **1**
P8 **Figure S13.** EI-MS spectrum of **1**.

P9 **Figure S14.** ^{1}H NMR spectrum of **3** at 600 MHz in $CDCl_3$.
P9 **Figure S15.** (+)HRESIMS spectrum of **3**
P10 **Figure S16.** EI-MS spectrum of **3**.

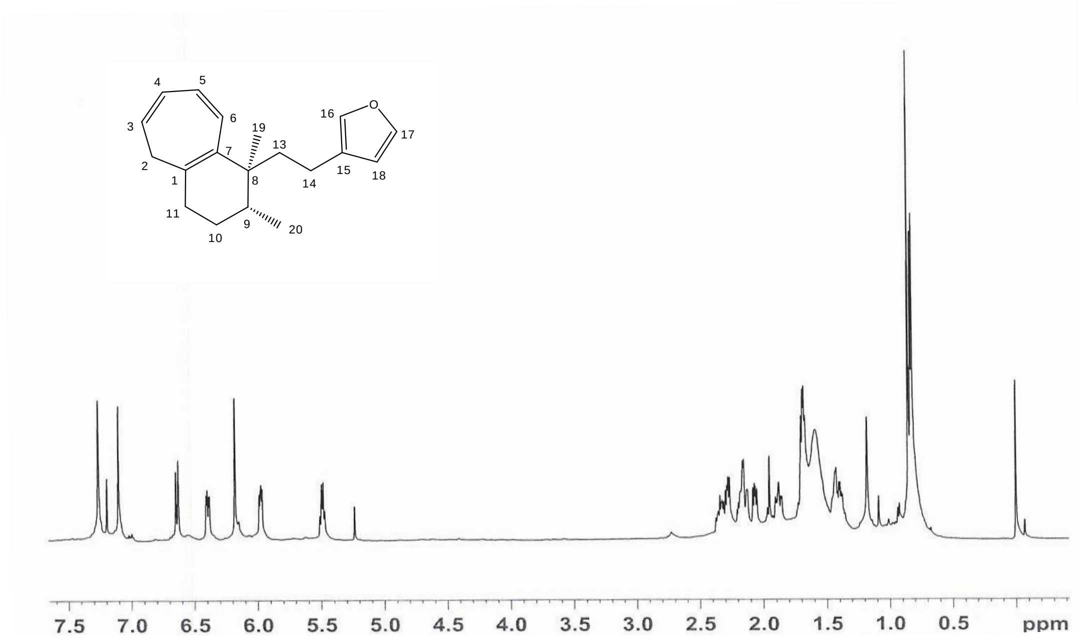

Figure S1. ^{1}H NMR spectrum of **1** at 600 MHz in $CDCl_3$.

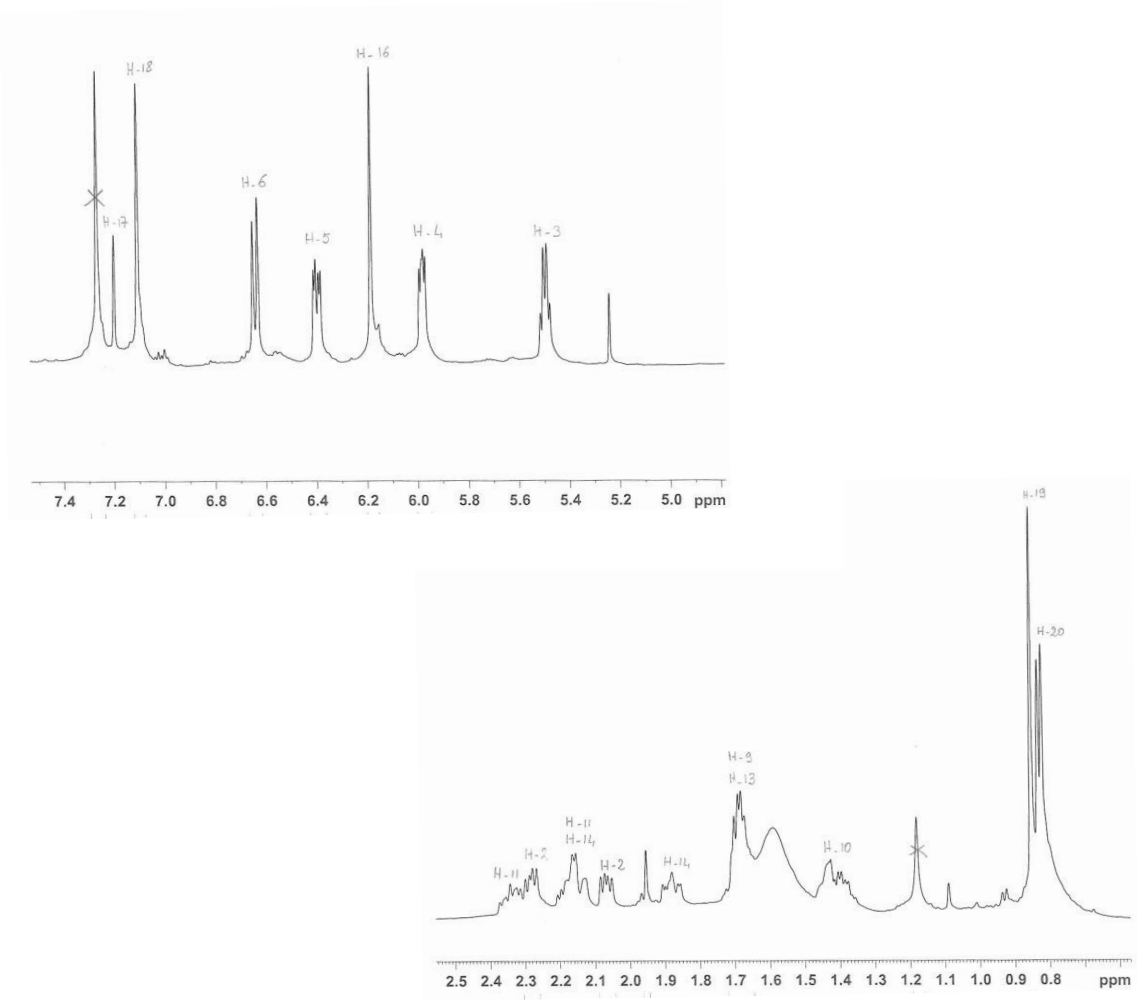

Figure S2. ^{1}H NMR spectrum of **1** at 600 MHz in $CDCl_3$.

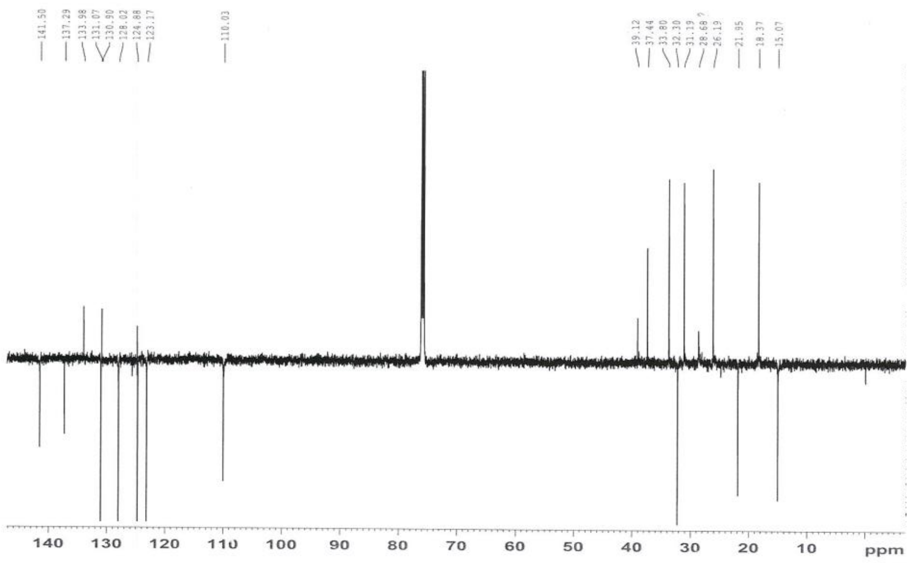

Figure S3. ^{13}C NMR (APT) spectrum of **1** at 150 MHz in $CDCl_3$.

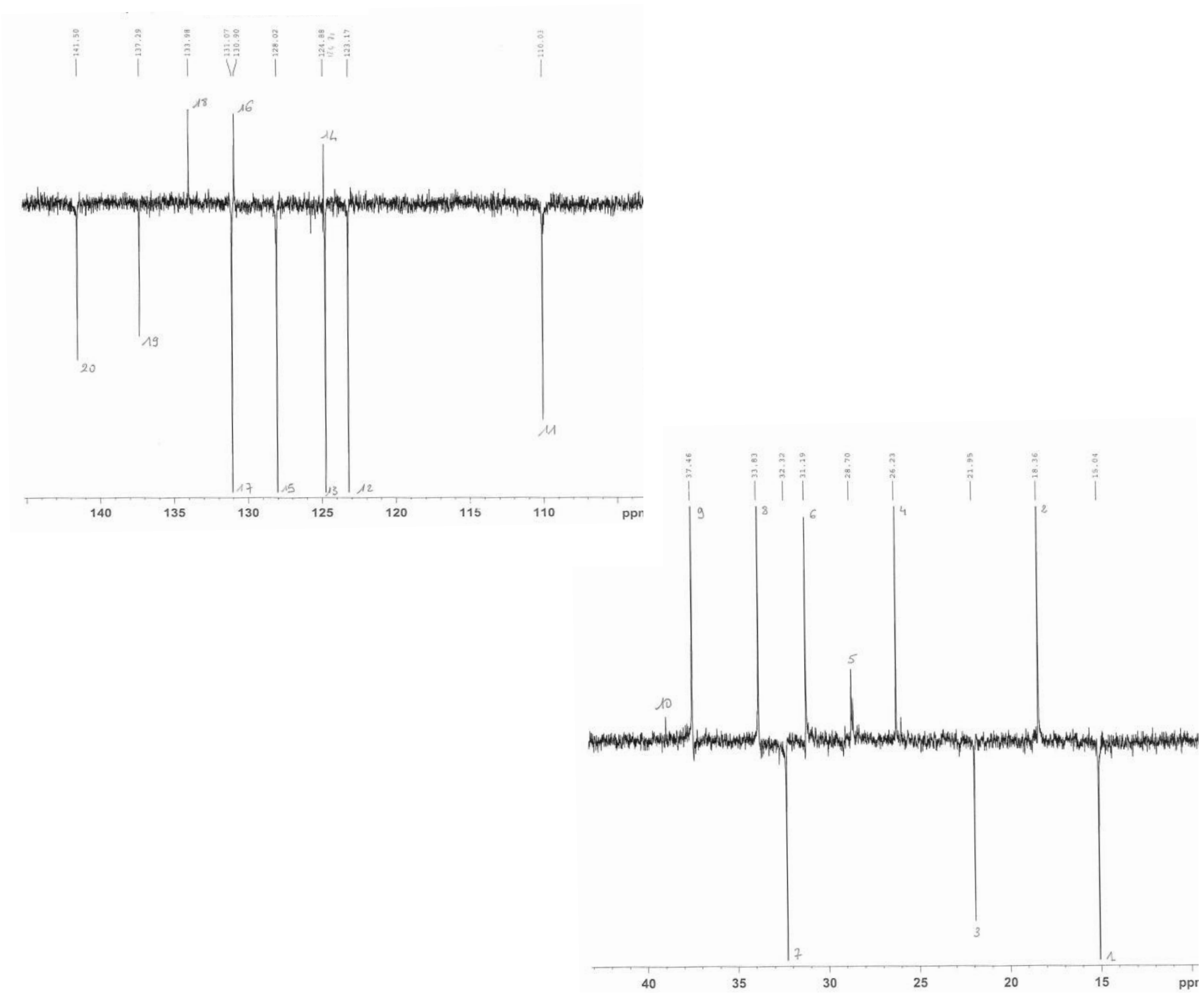

Figure S4. ^{13}C NMR (APT) spectrum of **1** at 150 MHz in $CDCl_3$.

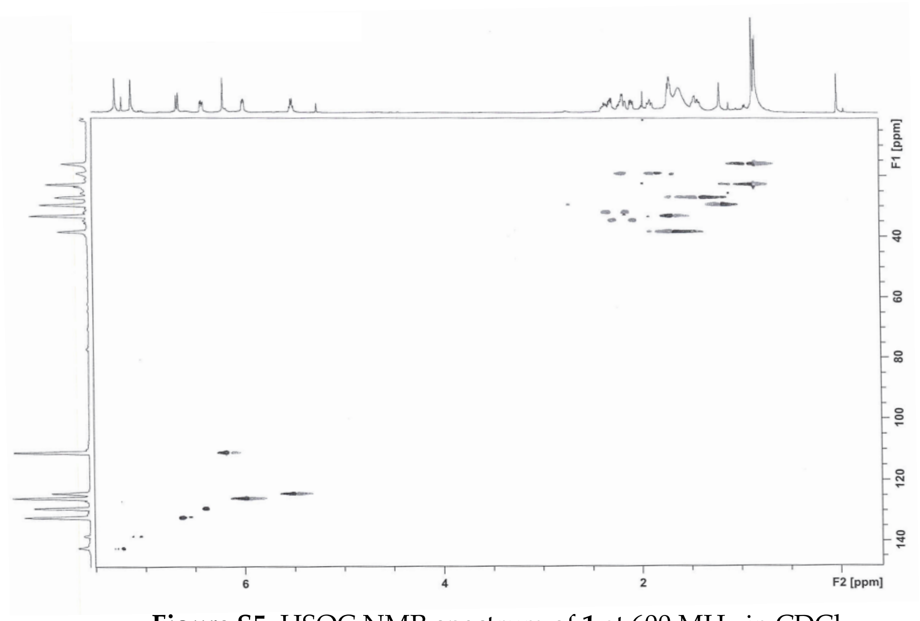

Figure S5. HSQC NMR spectrum of **1** at 600 MHz in $CDCl_3$.

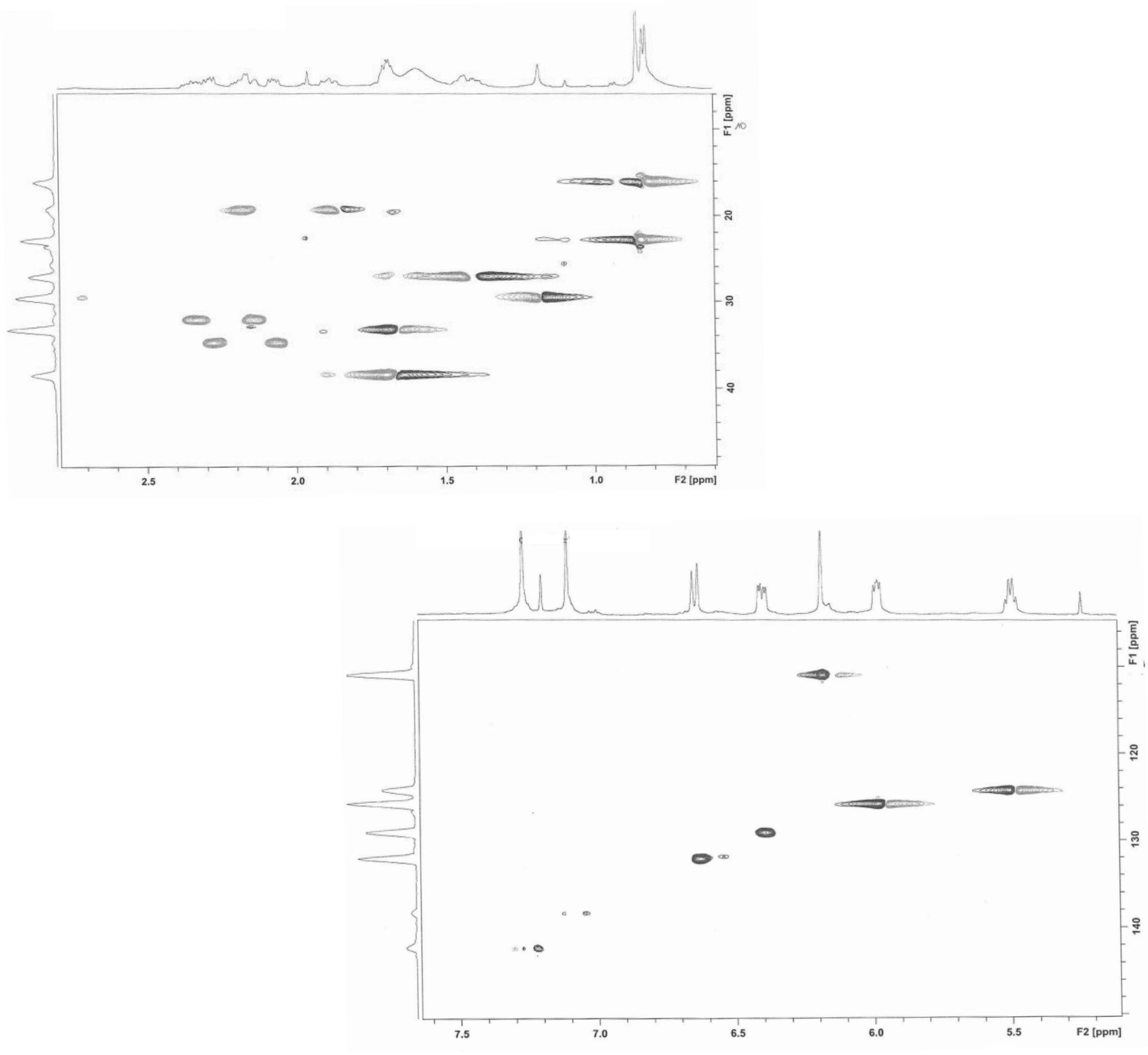

Figure S6. HSQC NMR spectrum of **1** at 600 MHz in $CDCl_3$.

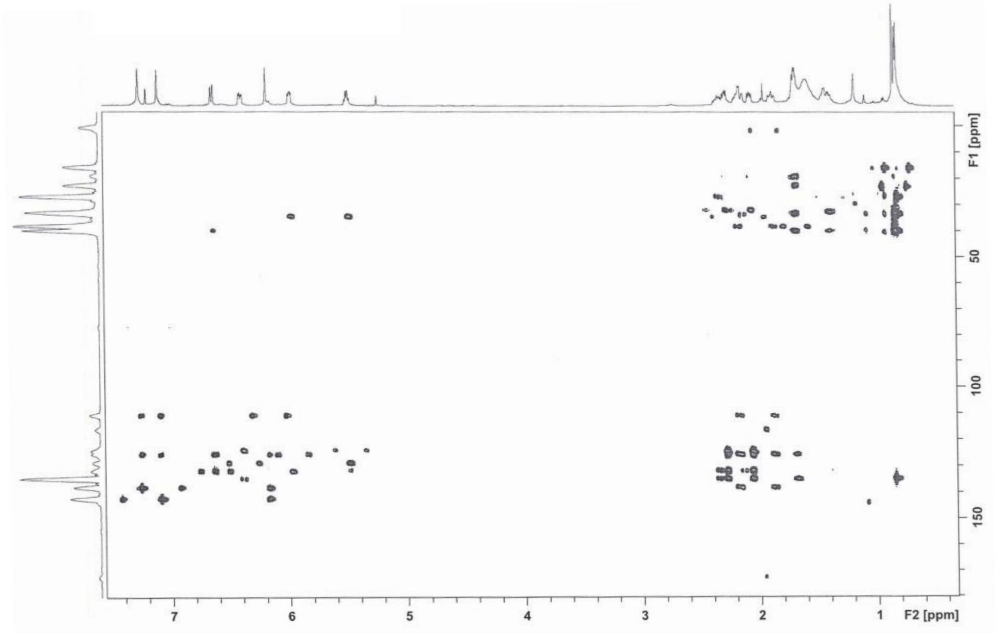

Figure S7. HMBC NMR spectrum of **1** at 600 MHz in $CDCl_3$.

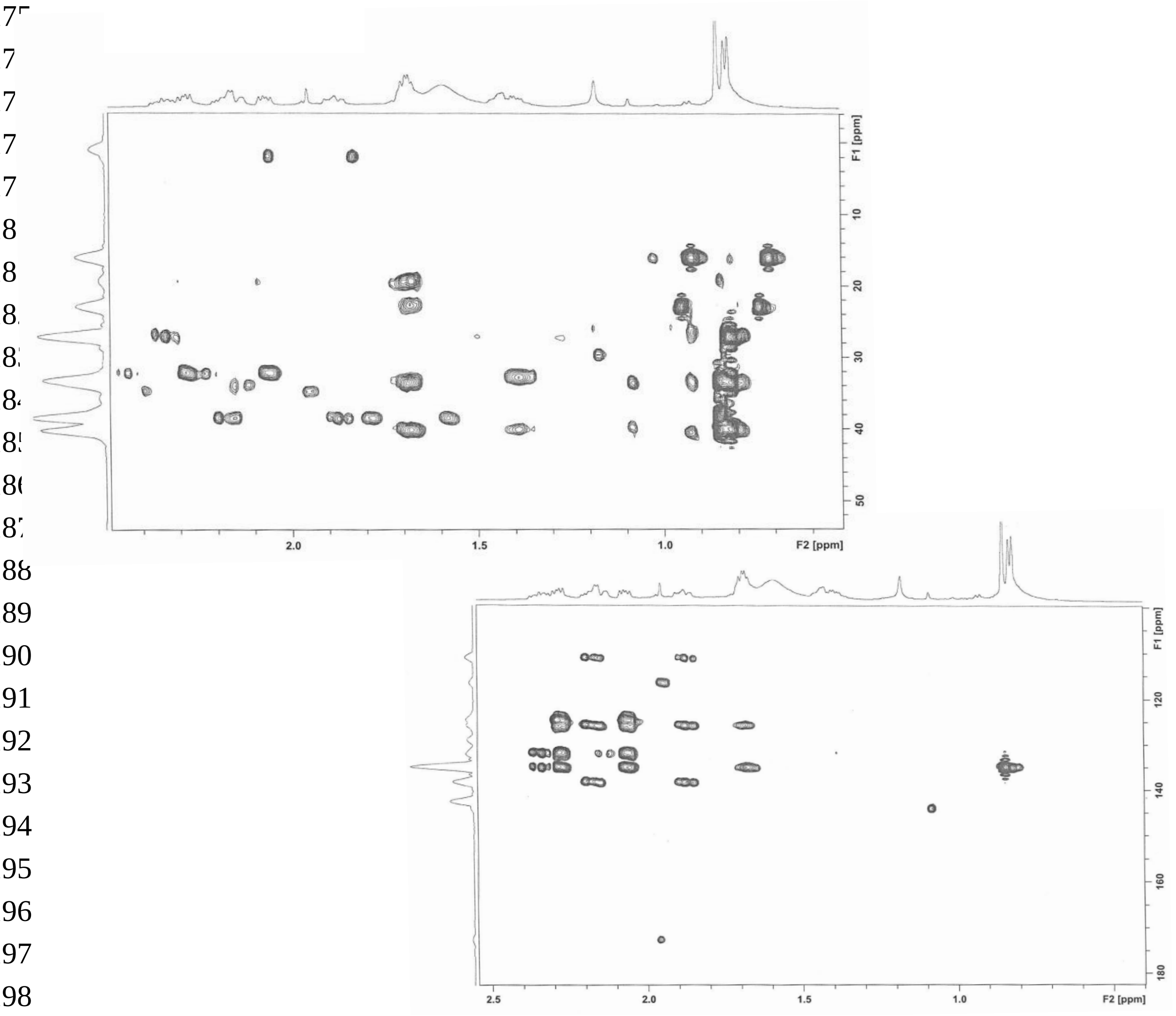

Figure S8. HMBC NMR spectrum of **1** at 600 MHz in $CDCl_3$.

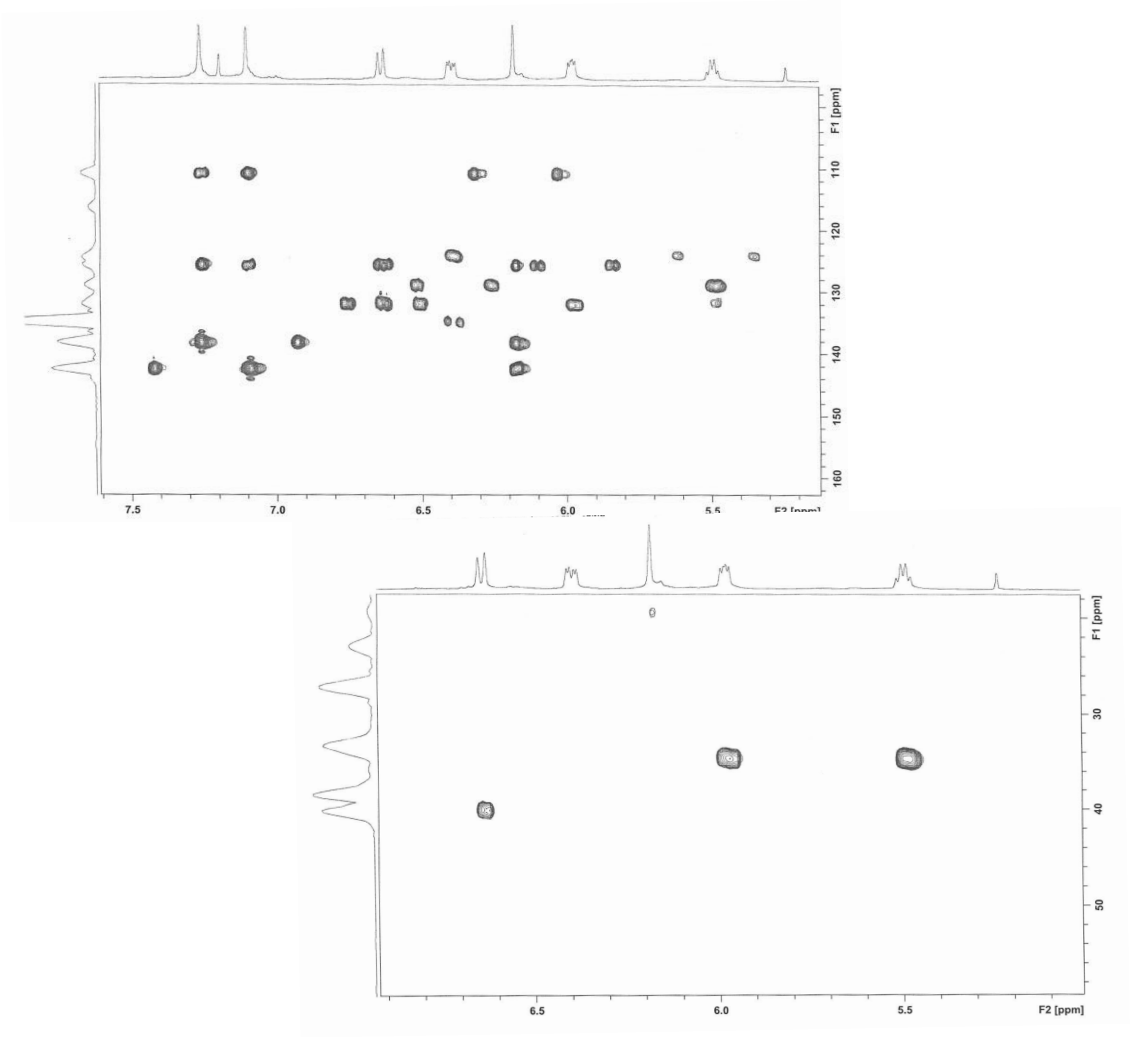

Figure S9. HMBC NMR spectrum of **1** at 600 MHz in $CDCl_3$.

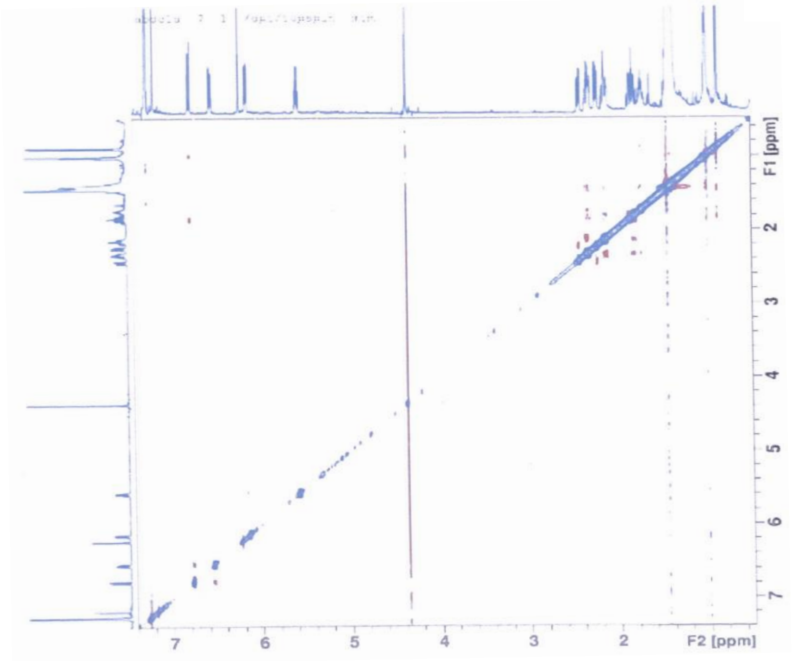

Figure S10. NOESY NMR spectrum of **1** at 600 MHz in C_6D_6.

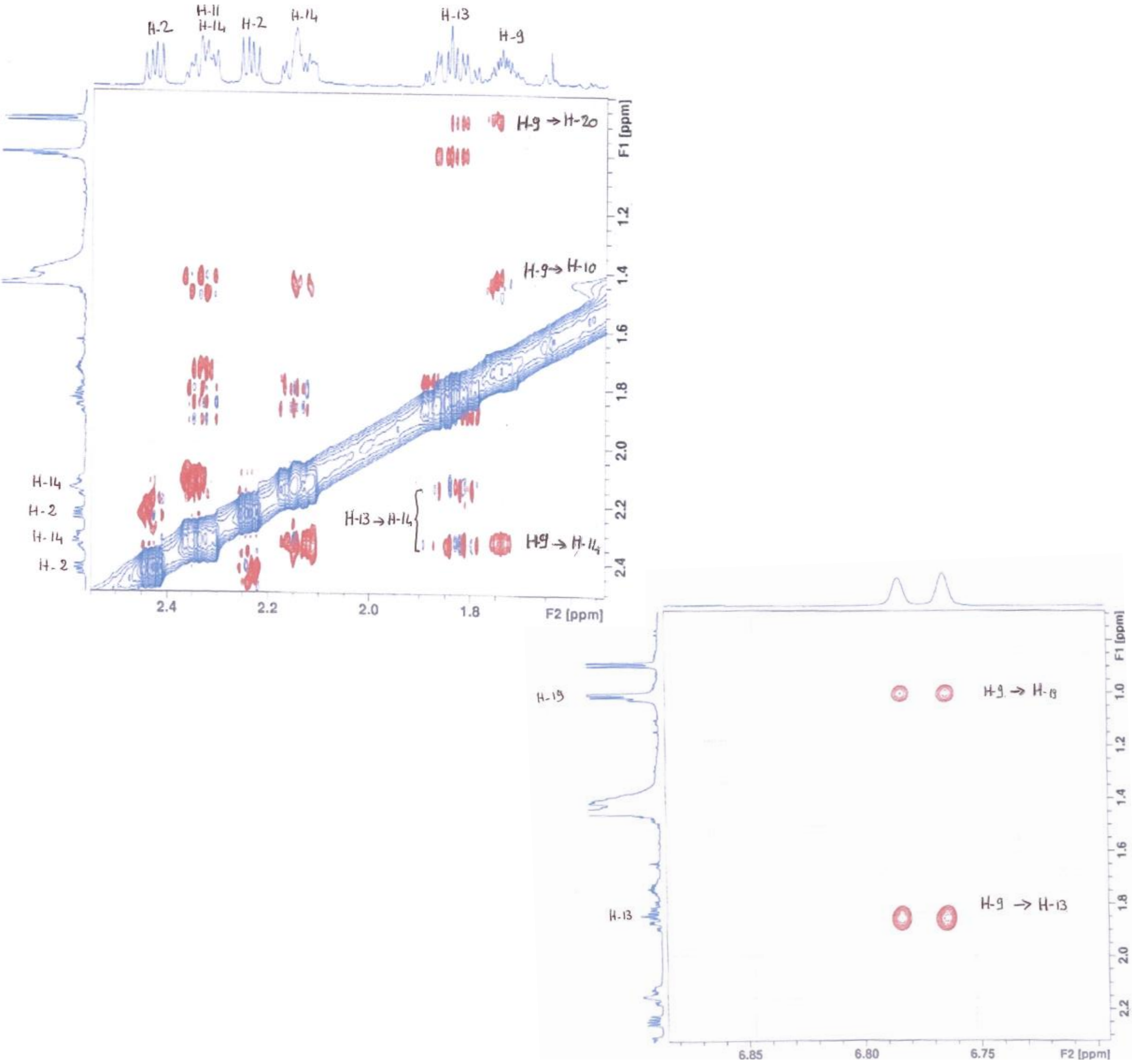

Figure S11. NOESY NMR spectrum of **1** at 600 MHz in C_6D_6.

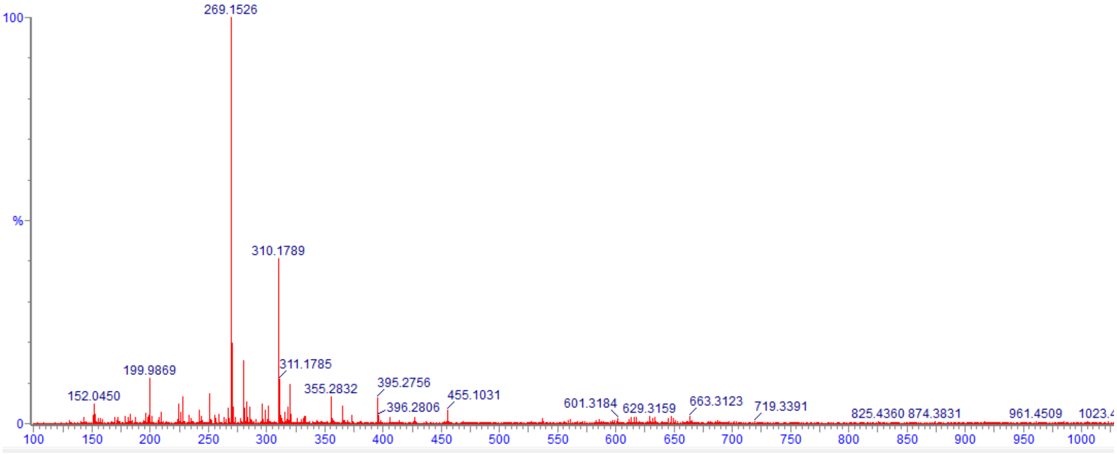

Figure S12. (+)HRESIMS spectrum of **1**

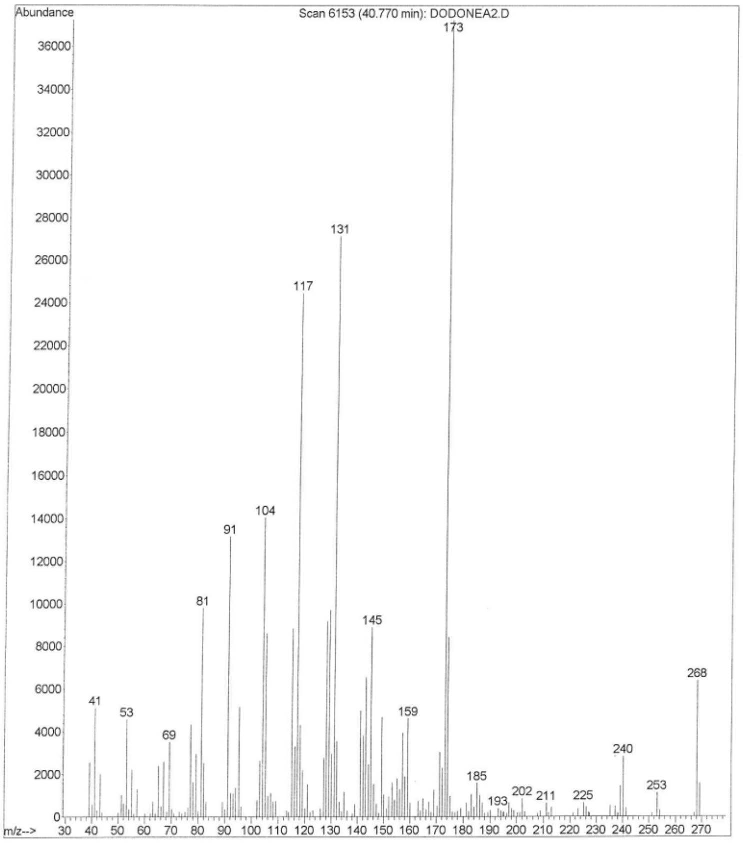

Figure S13. EI-MS spectrum of **1**.

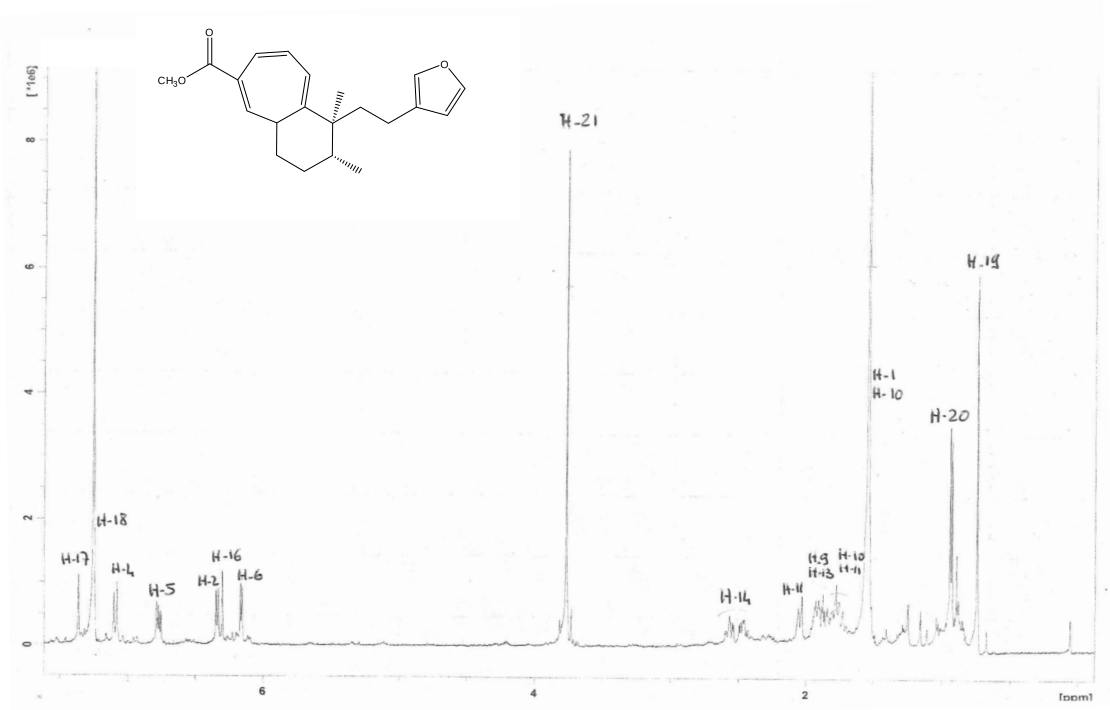

Figure S14. ^{1}H NMR spectrum of **3** at 600 MHz in $CDCl_3$.

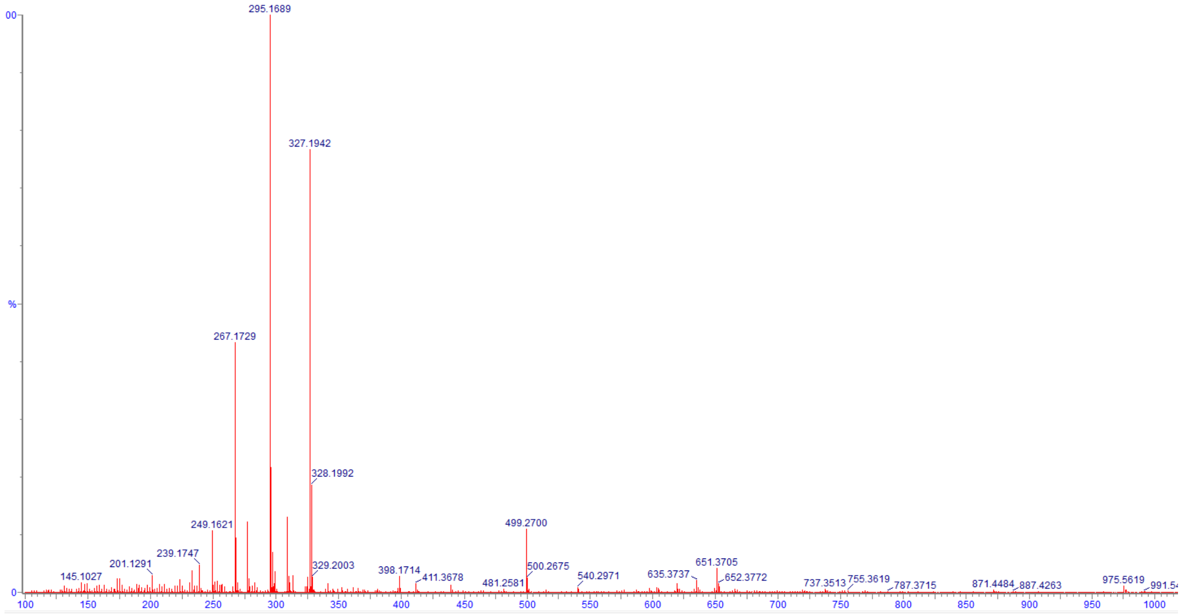

Figure S15. (+)HRESIMS spectrum of **3**

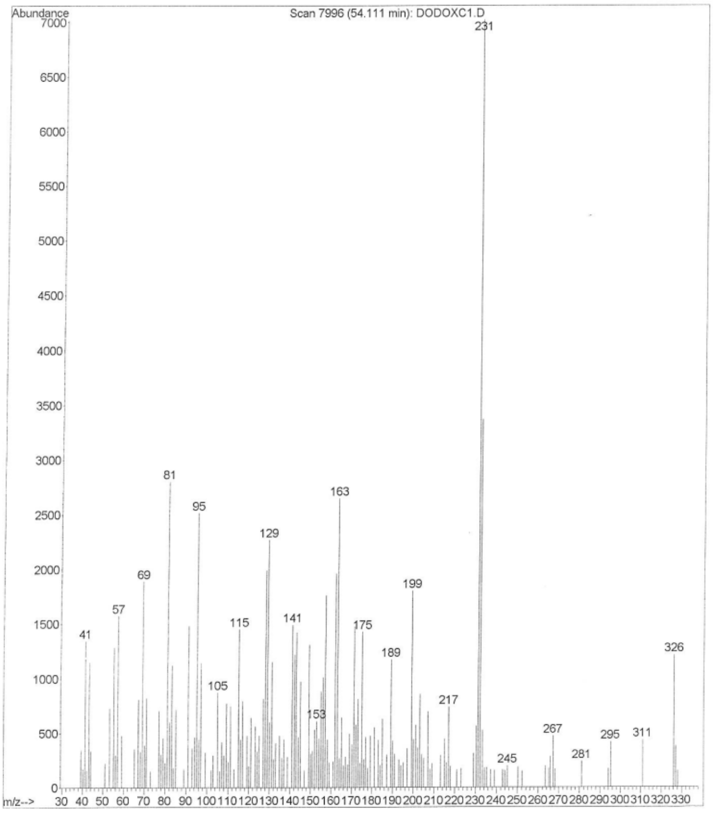

Figure S16. EI-MS spectrum of **3**.
